# Investigation of morphological changes for the discrimination of nucleated red blood cells and other leukocytes in Sysmex XN hematology analyzer scattergrams using transmission electron microscopy

**DOI:** 10.1016/j.plabm.2017.05.001

**Published:** 2017-05-10

**Authors:** Masako Kaido, Yuri Takagi, Mari Kono, Fumie Nakazawa, Shiori Yamamoto, Atsushi Wada, Takashi Morikawa

**Affiliations:** Scientific Research, Scientific Affairs, Sysmex Corporation, 1-3-2 Murotani, Nishi-ku, Kobe 651-2241, Japan

**Keywords:** WBCs, white blood cells, NRBCs, nucleated red blood cells, TEM, transmission electron microscopy, FCM, flow cytometer, FSC, forward scattered light, SFL, side fluorescence light, EDTA, ethylenediaminetetraacetic acid, FITC, fluorescein isothiocyanate, PE, phycoerythrin, MACS, magnetic cell sorting, MAS, Matsunami Adhesive Silane, Automated hematology analyzer, Flow cytometry, Transmission electron microscopy, Leukocytes, NRBCs, Scatter light intensity, Fluorescent intensity

## Abstract

**Background:**

The WNR channel of the XN-Series automated hematology analyzer (Sysmex) counts white blood cells (WBCs) and simultaneously performs a differential counting of basophils and nucleated red blood cells (NRBCs). The detection process involves exposing the cells to WNR-specific reagents containing an acidic detergent and a fluorescent dye and measuring the intensity of the forward scattered light (FSC) and side fluorescence light (SFL).

**Method:**

We treated isolated peripheral WBCs and NRBCs with specific reagents and assessed the morphological changes in NRBCs and each leukocyte type using transmission electron microscopy (TEM).

**Results:**

The results from a flow cytometer (FCM) showed that, after exposure to the reagents, basophils appeared on the highest FSC and SFL areas compared to other leukocytes on the WNR scattergram. Owing to the hemolysis of reticulocytes and erythrocytes, NRBCs that survived the reagent treatment could be distinguished by their lower intensity than those of the other leukocytes on the WNR scattergram. We investigated the significance of the relationship between the TEM and FCM results after the reagent treatment.

**Conclusion:**

We confirmed that the WNR channel differentiates the blood cells on the WNR scattergram based on differences in the amount of residual cytoplasm and nucleic acids.

## Highlights

• The WNR channel of the XN-Series counts WBCs and simultaneously performs a differential count of basophils and NRBCs.
• Basophils had the highest scattered light and fluorescent intensities after WNR treatment on a flow cytometer scattergram.
• NRBCs survived treatment with the WNR reagents had a lower intensity on the FCM scattergram than those of other leukocytes.
• TEM revealed that the WNR channel can differentiate blood cells based on the amount of residual cytoplasm and nucleic acids.

## 1 Introduction

Basophils constitute about 1–2% of circulating leukocytes. Basophil granules contain heparin and histamine. Basophilia occurs in chronic myelogenous leukemia and other myeloproliferative disorders, as well as some chronic inflammatory and infectious disorders [1]. Nucleated erythrocytes, NRBCs, are usually not present in peripheral blood of adults. During erythropoiesis, the process of erythroid maturation, there is progressive condensation of nuclear chromatin (termed *nuclear maturation*), which is eventually extruded from the cell [2], [3], [4]. NRBCs may appear in peripheral blood when the marrow is under intense stimulation such as for example due to haematological disorders, hemolysis, hemorrhage, or hypoxia. In patients with megaloblastic anemias, NRBCs in the blood have megaloblastic nuclear features [5], [6], [7]. In addition, NRBCs are also observed in cord blood and in the blood of fetuses and newborns [8], [9], [10], [11], [12].

In clinical laboratories, a set of qualification blood films using a wedge smear technique is prepared and microscopically reviewed by two skilled examiners. A 200-cell differential count is then performed manually under a microscope and any nucleated erythrocytes present are counted. The result is expressed as a proportion of the total 100 white blood cells (WBCs) counted [13]. This method is time-consuming, labor-intensive, and susceptible to discrepancies between examiners. Furthermore, an important limitation of the procedure is the statistical imprecision of the proportionate NRBC estimate due to the small number of cells counted. Thus, automated counting may be a beneficial alternative. However, it is difficult to distinguish NRBCs from leukocytes, particularly small lymphocytes, by common flow cytometer (FCM) based on electrical resistance and impedance measurements [14]. Recently, it has become more common to distinguish each type of blood cell using two-dimensional (2D) light scattergrams from an FCM based on treatment with a proper surfactant and staining dye [15]. The principle of flow cytometry prevents laboratory technicians from making errors that can induce variation and enables performance of many blood cell counts in a short period of time. Therefore, differential counting of basophils and NRBCs using an automated hematology analyzer has many potential benefits.

The XN-Series is a multi-parameter automated hematology analyzer from Sysmex. The WNR channel, which is one of the new functions of the XN-Series, counts WBCs and performs a differential count of basophils and NRBCs simultaneously based on this measurement principle. In the WNR detection process, a specific detergent in the Lysercell WNR perforates the cell membrane, causes hemolysis and dissolution of reticulocytes and mature erythrocytes that do not have a nucleus, and differentially disrupts the cytoplasmic membrane of NRBCs and WBCs. Then, a fluorescent dye in the Fluorocell WNR enters the cells and stains nucleic acids and organelles. The XN-Series measures the stained cell intensities as forward-scattered light (FSC; vertical axis), which represents cell size, and as side fluorescence light (SFL; horizontal axis), which represents the density of the intracellular contents. This information is subsequently expressed as a 2D scattergram, known as the WNR scattergram. On this scattergram, basophils have the highest FSC and SFL intensities and NRBCs the lowest SFL intensity compared to those of other leukocytes [16], [17], [18]. However, it is unclear why basophils and NRBCs appear in different areas of the scattergram from the other leukocytes. We analyzed the morphological differences between NRBCs from cord blood and lymphocytes, neutrophils, and basophils from healthy human peripheral blood using transmission electron microscopy (TEM) to investigate the reasons for their localization to different positions on the WNR scattergram. Specifically, we exposed blood cells to specific WNR reagents in a manner similar to that used for XN and analyzed them by general-purpose FCM and TEM.

## 2 Materials and methods

### 2.1 Samples

Peripheral blood was collected in EDTA-2K tubes (Terumo, Tokyo, Japan) from four healthy human volunteers who provided informed consent. In addition, four human umbilical cord blood donors from the Riken Bioresource center provided informed consent.

### 2.2 Cell preparation

The peripheral and umbilical cord blood samples were diluted in the same volume of phosphate-buffered saline (PBS). According to the manufacturer's instructions, blood was overlaid on 2 mL of Lymphocyte Separation Solution, with d =1.077 and d =1.119 (Nacalai Tesque, Inc., Kyoto, Japan). The leukocytes and NRBCs were concentrated by centrifugation at 100× *g* for 20 min; the platelet-rich plasma was removed and washed with PBS. After isolation, neutrophils, T lymphocytes, and basophils from the leukocyte fraction were separated using a magnetic cell sorting (MACS) system (RobSep; STEMCELL Technologies, Vancouver, BC, Canada) according to the manufacturer's instructions. Neutrophils, T lymphocytes, and basophils were incubated with fluorescein isothiocyanate (FITC)-conjugated mouse monoclonal antibodies to CD16b (neutrophil marker), CD3 (T lymphocyte marker), and CD123 (basophil marker). All antibodies were purchased from DAKO, Glostrup, Denmark, and were used at a concentration of 20 mg/L in PBS. The cells were incubated with the antibodies for 30 min at 4 °C in the dark. A FITC-conjugated mouse IgG1 antibody (DAKO, Glostrup, Denmark) was used as a negative control. The stained cells were then washed with PBS and analyzed using a FACSCalibur™ system (BD Biosciences, Franklin Lakes, NJ, USA).

### 2.3 XN measurement

Isolated cells from peripheral and umbilical cord blood were measured using Sysmex XN-2000 series multi-parameter automated hematology analyzers (Sysmex, Kobe, Japan) and their position on the WNR scattergram was investigated.

### 2.4 Scattergram analysis using general-purpose FCM

Exposure of peripheral blood to Lysercell WNR (Sysmex) and Fluorocell WNR (Sysmex) was performed in a manner similar to that used for XN, comprising 18 μL of sample, 1000 μL of Lysercell WNR, and 1 μL of Fluorocell WNR. Concentrated umbilical cord blood was stained with a phycoerythrin (PE)-conjugated mouse monoclonal anti-CD71 antibody (DAKO) at a concentration of 20 mg/L in PBS for 30 min at 4 °C in the dark, and then treated with Lysercell WNR and Fluorocell WNR in the same way as for peripheral blood for the XN system. A PE-conjugated mouse IgG1 antibody (DAKO) was used as a negative control. The stained cells were analyzed using a FACSCalibur™ system. The 2D scattergram was drawn with the FSC and SFL intensities on the vertical and horizontal axes, respectively, with adjustment for the detection range.

### 2.5 NRBC analysis

Umbilical cord blood including NRBCs concentrated by centrifugation was incubated with a PE-conjugated mouse monoclonal antibody to CD71 and a FITC-conjugated mouse monoclonal antibody to CD45 (leukocyte marker) (DAKO); the antibody was used at a concentration of 20 mg/L in PBS and the cells were incubated for 30 min at 4 °C in the dark. The CD71-positive and CD45-negative cell fractions were separated using an SH800 Cell Sorter (Sony Biotechnology Inc., Tokyo, Japan). A PE- or FITC-conjugated mouse IgG1 antibody (DAKO) was used as a negative control. The sorted fraction was treated with WNR reagents and analyzed using TEM.

### 2.6 TEM observation

All cells treated with WNR reagents were fixed with 1% glutaraldehyde (Electron Microscopy Sciences, Hatfield, PA, USA) solution in PBS for 16 h at 4 °C. The fixed cells were attached to Matsunami Adhesive Silane (MAS)-coated glass slides (Matsunami Glass Ind., Osaka, Japan) using Cytospin (Thermo Fisher Scientific, MA, USA) and post-fixed in osmium tetroxide for 45 min at room temperature. Following fixation, the cells were dehydrated in a graded series of ethanol and inverted-embedded in Quetol 812 (Nissin EM, Tokyo, Japan). The cells were cut into 70-nm thick sections with an Ultracut UCT Ultramicrotome (Leica Microsystems, Wetzlar, Germany) and observed under an H-7500 TEM (Hitachi High Technologies, Tokyo, Japan).

## 3 Results

### 3.1 The position of whole peripheral blood and NRBC-rich fractions of umbilical cord blood on the WNR scattergram was obtained using the XN-2000

**Fig. 1:**
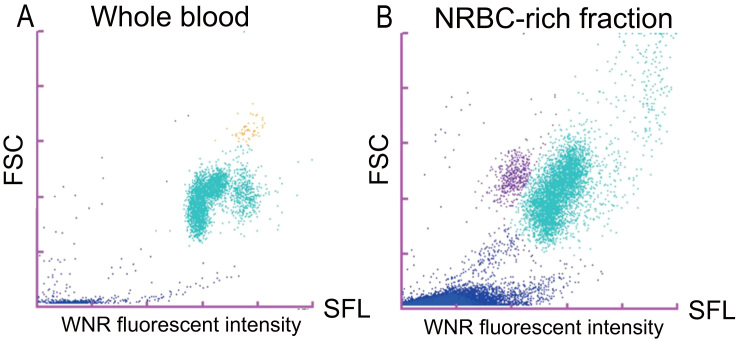
XN scattergram of healthy human whole peripheral blood and the NRBC-rich fraction obtained from umbilical cord blood. (A) On the WNR (SFL-FSC) scattergram of whole peripheral blood, the cells appear in the yellow-dotted area, which represents basophils, as well as in the turquoise-dotted area, which represents other leukocytes on the XN-2000. (B) In the NRBC-rich fraction, cells appear in the purple-dotted area, which represents NRBCs, and in the turquoise-dotted area, which represents other leukocytes on the XN-2000.

In whole peripheral blood, cells appearing in the yellow-dotted area of the XN 2000 analyzer were designated as basophils, while cells appearing in the turquoise-dotted area were designated as other leukocytes (Fig. 1A). In the NRBC-rich fraction, cells appearing in the purple-dotted area of the XN 2000 analyzer were designated as NRBCs, while cells appearing in the turquoise-dotted area were designated as other leukocytes (Fig. 1B).

### 3.2 Isolated leukocytes appear at the position of each leukocyte on the WNR scattergram

**Fig. 2:**
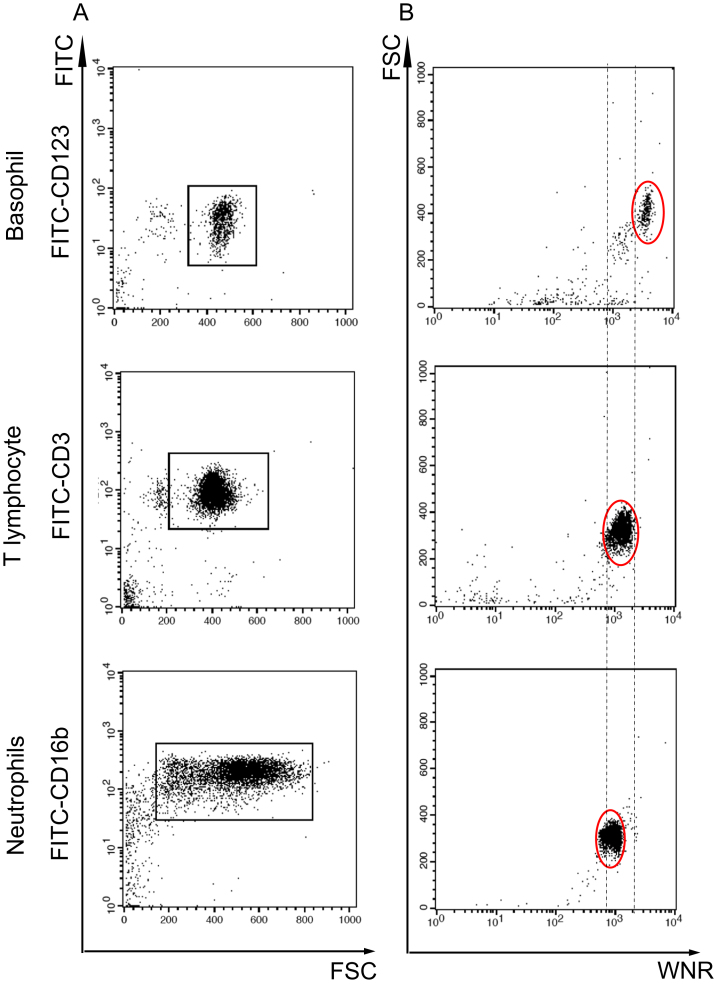
The positions of the three main subtypes of leukocytes isolated from whole peripheral blood by FACSCalibur. (A) Purity check of isolated leukocytes by FACSCalibur. Each subtype was stained with a specific anti-FITC-conjugated monoclonal antibody (basophils, anti-CD123; T lymphocytes, anti-CD3; neutrophils, anti-CD16b). The purities of the basophils, T lymphocytes, and neutrophils were 84.3%, 89.0%, and 92.0%, respectively. (B) Scattergram of the subtype fraction of each leukocyte isolated from whole blood after WNR reagent treatments. Upper, basophils; middle, T lymphocytes; lower, neutrophils. Isolated basophils had the highest FSC and SFL intensities.

Isolated neutrophils, T lymphocytes, and basophils had purities above 80% (Fig. 2A). After treatment with WNR reagents, each isolated leukocyte was measured using FACSCalibur and visualized on the WNR scattergram. Isolated basophils had the highest FSC and SFL intensities (Fig. 2B upper), which corresponded to the basophil area on the WNR scattergram (Fig. 1A yellow dots). T lymphocytes and neutrophils had lower FSC and SFL intensities than basophils (Fig. 2B middle, lower) and were located in the area classified as leukocytes on the WNR scattergram (Fig. 1A turquoise dots).

### 3.3 CD71-positive cells appear at the NRBC position on the WNR scattergram

**Fig. 3:**
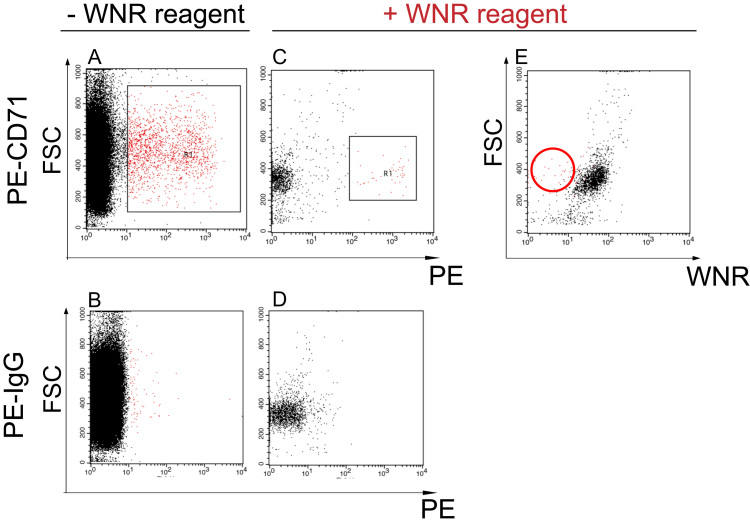
Scattergram of NRBC by FACSCalibur. The NRBC-rich fraction from cord blood was stained with a PE-conjugated negative control (B, D) or anti-CD71 antibody (A, C, E), double-stained with WNR-specific reagents (C-E), and analyzed by FACSCalibur. (A, B) PE-positive cells appear on the right part of the scattergram as CD71-positive reticulocytes and NRBCs (red dots in square). (C, D) The number of total cells and CD71-positive cells (red dots) decreased with use of WNR-specific reagents. The CD71-positive cells were gated (red dots in square). (E) Representation of the WNR scattergram by FACSCalibur. The SFL intensity of the CD71-positive cells (red dots) is lower than that of the CD71-negative cells (black dots) and appears in the expected area (red circle), which represents NRBC on the WNR scattergram.

First, a PE-positive cell cluster appeared on the right side of the scattergram as CD71-positive reticulocytes and NRBCs after anti-CD71 antibody single-staining (Fig. 3A red dots, B). Next, after treatment with WNR reagents, the number of total cells (Fig. 3A black dots, 3C black dots) and CD71-positive cells decreased (Fig. 3A black square, 3C black square). After gating the remaining CD71-positive cells (3 C black square), the FSC intensity was plotted on the vertical axis and the SFL WNR fluorescence was re-plotted on the horizontal axis. The SFL intensity of the CD71-positive cells (Fig. 3E red circle) was lower than that of the CD71-negative cells (Fig. 3C black dots) and appeared in the expected area, which is designated as NRBCs on the WNR scattergram (Fig. 1B).

### 3.4 The effect of WNR-specific reagents on morphological changes in blood cells

**Fig. 4:**
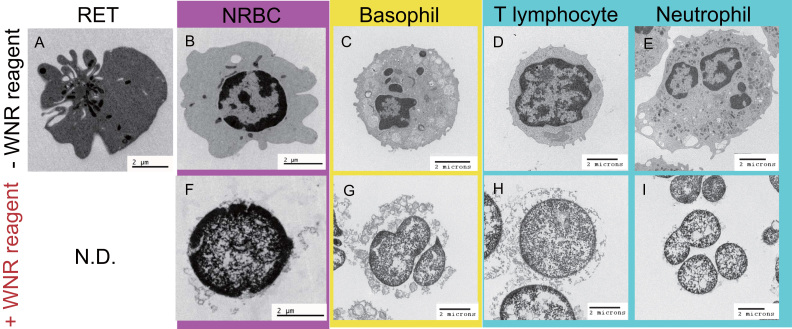
Transmission electron microscopy images of each cell treated with WNR-specific reagents. Upper (A-E): Prior to WNR-specific reagent treatment, the cells showed specific morphological properties. Lower (F-I): After treatment, the basophils retained their cell morphology, whereas other leukocytes and erythroblasts showed extensive damage. The level of residual internal structures observed via TEM was reflected in the SFL intensity on the WNR scattergram.

Prior to WNR treatment, the leukocytes showed specific morphological properties (Fig. 4C-E), as previously reported [18], [19]. Both nucleated erythrocytes and reticulocytes were observed in the CD71-positive and CD45-negative erythroblast fractions (Fig. 4A, B). After treatment with the detergent, reticulocytes were not detected due to the destruction of the cells, also known as hemolysis. However, TEM revealed that the basophils retained most of their cytoplasm, while lymphocytes retained part of their cytoplasm. In contrast, neutrophils and CD71-positive nucleated cells lost most of the cytoplasm and retained their nucleus (Fig. 4F-I).

## 4 Discussion

The high FSC and SFL intensities of basophils (Fig. 2B) are reflected by the observations of WNR fluorescent intensity in confocal laser scanning microscopy [18]. The decreasing number of CD71-positive and negative cells after WNR reagent treatment suggested that reticulocytes and erythrocytes disappeared due to hemolysis. Moreover, the absence of both types of cells in TEM suggested that CD71-positive erythroblasts and CD71-negative leukocytes are retained after WNR reagent treatment (Fig. 3A, C). We then confirmed that CD71-positive erythroblasts had a lower SFL intensity than that of the CD71-negative leukocytes on the scattergram (Fig. 3E red circle). Next, we assessed the morphological properties of each cell using TEM to determine why different types of blood cells appeared in different positions. TEM observation of each type of leukocyte after WNR-specific reagent treatment revealed that basophils and T lymphocytes retained most and part of their cytoplasm, respectively, whereas neutrophils lost most of the cytoplasm and retained only their nucleus (Fig. 4G-I). This observation indicates that the intensities of WNR fluorescence on the WNR scattergram reflected the level of residual internal structures of each leukocyte. In contrast, the TEM observation of CD71-positive cells after WNR-specific reagent treatment revealed that CD71-positive nucleated cells, which must be NRBCs, lost most of their cytoplasm and seemed to retain their nucleus, similar to neutrophils (Fig. 4F, 4I). This observation suggested that the position of NRBCs on the WNR scattergram was the same as that of neutrophils after the WNR reagent reactions, mainly because the reagent stained nucleic acids. In this validation process, the FSC intensities of NRBCs and leukocytes were the same (Figs. 1B, 2B), but the WNR fluorescence intensity of the NRBCs was lower than that of leukocytes; thus, NRBCs could be differentiated from leukocytes (Figs. 1B, 3E). In the process of erythrocyte differentiation, a reduced capacity to incorporate amino acids was demonstrated in the nuclear expulsion, as was the disaggregation of the polysomes observed in TEM [20]. Moreover, as actinomycin D did not prevent the expulsion of the nucleus from the late stages of the erythroid line, new mRNA was not thought to be required for instructing the cell to expel its nucleus. Therefore, the total amount of RNA in the NRBC nucleus decreased and the WNR fluorescence intensity of NRBCs on the WNR scattergram was lower than that of leukocytes due to RNA depletion. In addition to this biological explanation, this observation can be explained by the fact that NRBCs are more susceptible to the reagent than lymphocytes and thus lose more RNA during the treatment.

We confirmed that the WNR channel differentiates blood cells on the WNR scattergram based on differences in the amount of residual cytoplasm and nucleic acids.

## Conflict of interests

The authors have declared no conflicts of interest.
